# X-linked serotonin 2C receptor is associated with a non-canonical pathway for sudden unexpected death in epilepsy

**DOI:** 10.1093/braincomms/fcab149

**Published:** 2021-07-09

**Authors:** Cory A Massey, Samantha J Thompson, Ryan W Ostrom, Janice Drabek, Olafur A Sveinsson, Torbjörn Tomson, Elisabeth A Haas, Othon J Mena, Alica M Goldman, Jeffrey L Noebels

**Affiliations:** 1 Developmental Neurogenetics Laboratory, Department of Neurology, Baylor College of Medicine, Houston, TX 77030, USA; 2 Department of Neurology, National University Hospital of Iceland, 101 Reykjavik, Iceland; 3 Department of Clinical Neuroscience, Karolinska Institutet, Stockholm 171 76, Sweden; 4 Department of Pathology, Rady Children’s Hospital-San Diego, San Diego, CA 92123, USA; 5 Medical Examiner Office, Ventura County Health Care Agency, Ventura, CA 93003, USA; 6 Department of Neuroscience, Baylor College of Medicine, Houston, TX 77030, USA; 7 Department of Human and Molecular Genetics, Baylor College of Medicine, Houston, TX 77030, USA

**Keywords:** SUDEP, HTR2C, 5-HT, sex differences, GABA

## Abstract

Sudden Unexpected Death in Epilepsy is a leading cause of epilepsy-related mortality, and the analysis of mouse Sudden Unexpected Death in Epilepsy models is steadily revealing a spectrum of inherited risk phenotypes based on distinct genetic mechanisms. Serotonin (5-HT) signalling enhances post-ictal cardiorespiratory drive and, when elevated in the brain, reduces death following evoked audiogenic brainstem seizures in inbred mouse models. However, no gene in this pathway has yet been linked to a spontaneous epilepsy phenotype, the defining criterion of Sudden Unexpected Death in Epilepsy. Most monogenic models of Sudden Unexpected Death in Epilepsy invoke a failure of inhibitory synaptic drive as a critical pathogenic step. Accordingly, the G protein-coupled, membrane serotonin receptor 5-HT_2C_ inhibits forebrain and brainstem networks by exciting GABAergic interneurons, and deletion of this gene lowers the threshold for lethal evoked audiogenic seizures. Here, we characterize epileptogenesis throughout the lifespan of mice lacking X-linked, 5-HT_2C_ receptors (loxTB Htr2c). We find that loss of *Htr2c* generates a complex, adult-onset spontaneous epileptic phenotype with a novel progressive hyperexcitability pattern of absences, non-convulsive, and convulsive behavioural seizures culminating in late onset sudden mortality predominantly in male mice. RNAscope localized *Htr2c* mRNA in subsets of *Gad2*+ GABAergic neurons in forebrain and brainstem regions. To evaluate the contribution of 5-HT_2C_ receptor-mediated inhibitory drive, we selectively spared their deletion in GAD2+ GABAergic neurons of pan-deleted loxTB Htr2c mice, yet unexpectedly found no amelioration of survival or epileptic phenotype, indicating that expression of 5-HT_2C_ receptors in GAD2+ inhibitory neurons was not sufficient to prevent hyperexcitability and lethal seizures. Analysis of human Sudden Unexpected Death in Epilepsy and epilepsy genetic databases identified an enrichment of *HTR2C* non-synonymous variants in Sudden Unexpected Death in Epilepsy cases. Interestingly, while early lethality is not reflected in the mouse model, we also identified variants mainly among male Sudden Infant Death Syndrome patients. Our findings validate *HTR2C* as a novel, sex-linked candidate gene modifying Sudden Unexpected Death in Epilepsy risk, and demonstrate that the complex epilepsy phenotype does not arise solely from 5-HT_2C_-mediated synaptic disinhibition. These results strengthen the evidence for the serotonin hypothesis of Sudden Unexpected Death in Epilepsy risk in humans, and advance current efforts to develop gene-guided interventions to mitigate premature mortality in epilepsy.

## Teaser

Massey et al. report that mice lacking serotonin 2C receptors are an adult-onset model of sudden unexpected death in epilepsy (SUDEP). Sparing this deletion in interneurons reveals that the complex epilepsy phenotype is not due solely to disinhibition. They also report 5-HT_2C_ variants in cases of human SUDEP.

## Graphical Abstract

**Graphical Abstract:**
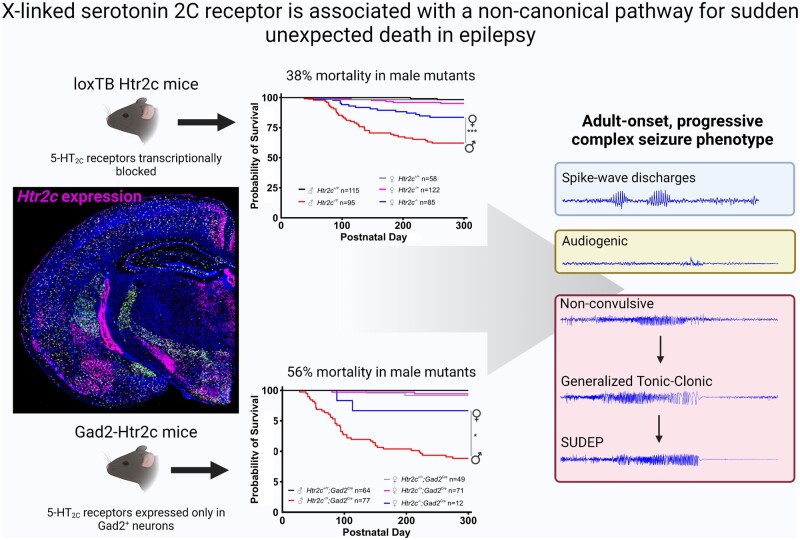

## Introduction

Sudden Unexpected Death in Epilepsy (SUDEP) is a leading cause of mortality in individuals with epilepsy, accounting for nearly 3000 deaths per year in the U.S.^1–3^ In patients with chronic refractory epilepsy, SUDEP is diagnosed as the cause of death in up to 50% of cases.^4^^,^^5^ The risk of sudden death is 20 times higher in patients with epilepsy compared to the general population and can be stratified according to clinical and genetic factors.^6^ Monogenic epilepsies, such as Dravet Syndrome (*SCN1A*), provide additional insight into the heterogeneity of SUDEP mechanisms in paediatric epilepsy populations, as well as an opportunity to more precisely identify and treat individuals at increased risk.^7–9^ Mouse models of these and related genes have been identified that significantly raise early SUDEP risk by impairing cardiorespiratory function following a tonic seizure,^10–16^ while few genes for adult-onset risk have been reported. Analysis of these early death models indicates the critical importance of defective synaptic inhibition. For example, in heterozygous *Scn1a* mutants, selective activation of inhibitory neurons by CRISPR-Cas9 engineering^17^ or a specific venom peptide toxin^18^ pinpoint the impairment of interneuron firing as a critical mechanism underlying seizures and SUDEP.

The neurotransmitter serotonin (5-hydroxytryptamine; 5-HT) is implicated in a number of neuropsychiatric disorders, including epilepsy, and the ‘serotonin hypothesis’ frames a clear mechanistic pathway contributing to SUDEP.^19^^,^^20^ 5-HT signalling is anticonvulsant and decreases seizure susceptibility in different experimental models.^21–23^ Faingold et al.^24^^,^^25^ have shown that seizure-induced respiratory arrest and death following audiogenic seizures (AGSs) in inbred DBA/1 and DBA/2 mice can be prevented by pretreatment with the selective serotonin reuptake inhibitor, fluoxetine, or optogenetic stimulation of 5-HT neurons in the dorsal raphe. Moreover, expression of multiple 5-HT receptors, including 5-HT_2C_, is decreased in the brainstem of these mice compared to C57/Bl6 mice.^26^ 5-HT_2C_ (formerly known as 5-HT_1C_) is a 5-HT metabotropic receptor expressed by the X-linked gene *HTR2C* throughout the CNS, including retrosplenial and piriform cortex, amygdala, subthalamic nucleus, lateral habenula and brainstem.^27–31^ These receptors are predominantly coupled to G_q/11_ proteins and activate phospholipase C to release intracellular Ca^2+^ stores, thereby altering excitability.^32^

5-HT_2C_ receptors reside predominantly on GABAergic neurons, and their activation by 5-HT increases spontaneous activity of these interneurons.^33^^,^^34^ A 5-HT_2C_ knockout model revealed that null male mice display AGS and increased mortality (38%) beginning at 2–4 months of age compared to wildtype littermates, presumably due to network disinhibition.^35^ A second knockout model in which a floxed transcriptional blocker was inserted between exons three and four of the *Htr2c* gene (loxTB Htr2c) also showed behavioural convulsions and reduced survival,^36^ however, neither of these models were analysed by EEG monitoring.

Here, we investigated the epileptic phenotype of the loxTB Htr2c mouse line and discovered that 5-HT_2C_ mutant mice present a hitherto unreported syndrome of multiple adult-onset seizure types with a clear predominance of SUDEP in male mice. We then used a mouse line that expressed Cre recombinase under the control of *Gad2* to excise the transcription block in loxTB Htr2c mice, preserving the expression of 5-HT_2C_ receptors only in GAD2-positive cells. We studied this selective GAD2+ neuronal expression model to determine whether the loss of 5-HT_2C_ receptors in a major population of GABAergic interneurons is the predominant driver of seizures and premature mortality in 5-HT_2C_ mutant mice. In addition, we report here the first analysis of *HTR2C* variants found in human SUDEP and sudden infant death syndrome (SIDS) cohorts.

## Methods

### Animals

All experiments were approved by the Baylor College of Medicine Institutional Animal Care and Use Committee. Mice were housed in groups of one to five animals in a temperature- and humidity-controlled room with a 14-h light and 10-h dark cycle, with access *ad libitum* to water and food. loxTB Htr2c mice were a gift of the Elmquist and Xu laboratories.^36^ These mice have a transcriptional blocker flanked by loxP sites (loxTB) inserted between exons three and four of the X-linked *Htr2c* gene, which prevents transcription of 5-HT_2C_ receptors. These mice are functional knockouts, and we refer to male and female 5-HT_2C_-null mice as *Htr2c*^*−*^^*/Y*^ and *Htr2c*^*−*^^*/*^^*−*^, respectively. We obtained male mice expressing Cre recombinase under the control of *Gad2* (B6J.Cg-*Gad2^tm^*^2^^*(cre)Zjh*^/MwarJ; Stock #028867) from Jackson Labs and bred them to female *Htr2c*^*−*^^*/+*^ mice. This cross produced mice with the transcriptional blocker excised in only *Gad2*-positive neurons (Gad2^Cre^-Htr2c).

### Survival analysis

We tracked mice up to 41–42 weeks old and counted deaths in the home cage during this period. Any animal requiring euthanasia per veterinary guidelines was excluded from the study. When possible, we documented the body position of deceased animals, and those that showed post-mortem hindlimb extension and a pronounced hunched back were considered probable SUDEP cases.

### Video-EEG monitoring

Video-EEG recordings were performed as previously described.^37^ All surgical procedures were performed in a Baylor Center for Comparative Medicine surgery facility. Prior to surgery, mice were weighed and treated with meloxicam (5 mg/kg, s.c.) for analgesia. Mice were anesthetized with 2.5% isoflurane using a Patterson Scientific isoflurane vaporizer, and the scalp prepped using aseptic technique. Insulated silver EEG leads pre-wired to a nano strip connector (Omnetics Connector Corporation, Minneapolis, MN) were implanted in wildtype and mutant littermates through bilateral cranial burr over frontal and parietal cortex with a ground and reference electrode over the olfactory bulbs. Following surgery, mice were returned to their home cage and treated with a daily meloxicam (5 mg/kg, s.c.) injection for 3 days. Implanted mice were allowed 10–14 days post-operative recovery in their home cage before recordings began. EEG in freely behaving mice was recorded with a USBamp biosignal amplifier (g.tec USA, Albany, NY), digitized using a PowerLab 16/35 system (AD Instruments, Inc., Colorado Springs, CO), and analysed with LabChart 8 Pro software (AD Instruments, Inc.). A band-pass digital filter was applied during EEG analysis with a high cut-off frequency of 50 Hz and a low cut-off frequency of 0.3 Hz. Additional recording criteria can be found in Supplementary material.

### AGS induction

A previous study reported that 5-HT_2C_-null mice are susceptible to AGS.^38^ To assess EEG during these sound-invoked convulsions, video-EEG recordings were performed for 12–48 h to obtain a baseline prior to AGS induction. Following the baseline period between the hours of 1–3 p.m. Central Time, animals were exposed to a 107 dB broadband acoustic tone generated by an analog twin bell alarm (La Crosse Technology, La Crosse, Wisconsin) placed in the recording cage until the appearance of convulsive movements or for a maximum of 60 s.

### Htr2c expression

We determined regional expression levels of *Htr2c* mRNA in loxTB Htr2c and Gad2^Cre^-Htr2c mice by quantitative polymerase chain reaction (qPCR). Adult mice [postnatal day (PD) >45] were deeply anesthetized with isoflurane (>4%) and then decapitated. We harvested a 2 mm slab of coronal forebrain located 4 mm from the anterior pole that contained the majority of cortical and subcortical regions expressing *Htr2c* (dorsal hippocampus, habenula, thalamus, hypothalamus) which was flash frozen with dry ice. Next, we extracted RNA from this tissue using the RNAeasy Plus Mini Kit (Qiagen, Germantown, MD) and assayed RNA concentration using a NanoDrop One (Thermo Scientific, Waltham, MA). We used 200 ng of RNA material in the High Capacity cDNA Reverse Transcription Kit (Applied Biosystems, Foster City, CA) to generate cDNA for analysis. TaqMan probes targeting mouse *Htr2c* (Mm00434127_m1; Applied Biosystems) and *Gapdh* (Mm99999915_g1; Applied Biosystems) with TaqMan Fast Advanced Master Mix (Applied Biosystems) were used to assess gene expression. Samples were loaded on 96-well plates, and qPCR analysis was performed on an Applied Biosystems QuantStudio 3 system. Age-matched wildtype and mutant samples were assayed in triplicate, and male and female samples were assayed on separate plates. For a more detailed description of our qPCR analysis, see Supplementary material.

### Cellular localization of *Htr2c* in mouse brain

Brains were extracted from wildtype mice and divided into forebrain and midbrain/hindbrain samples with a cut at the level of the superior colliculus. Each of these were hemisected by a midsagittal cut. Immediately following extraction, the right-sided samples were placed in a brain mold, covered in Optimal Cutting Temperature embedding medium, and placed on dry ice to preserve RNA quality. Tissue sections (of rostral and caudal blocks) were cryo-sectioned using a CM1950 cryostat (Leica Biosystems, Buffalo Gove, IL) into 15 µm thick hemi-coronal sections and mounted onto Superfrost Plus slides, then stored at −80°C until use.

RNAscope Multiplex Fluorescent V2 kit was used to perform RNAscope hybridization (Advanced Cell Diagnostics, Newark, CA). For our experiments, we used channel one probes targeting *Gad2* (Mm-Gad2; Cat. no. 439371; Advanced Cell Diagnostics), channel two probes targeting *Htr2c* (Mm-Htr2c-C2; Cat. No. 401001-C2; Advanced Cell Diagnostics) and channel three probes targeting *Slc32a1* (Mm-Slc32a1-C3; Cat. No. 319191-C3; Advanced Cell Diagnostics) mRNA species. Channel two and three probes were diluted into channel one probes at a 1:1:50 ratio. Probes were labelled using Opal fluorescent dyes (Akoya Biosciences, Marlborough, MA) at a 1:1500 dilution. We used Opal dye 520 for channel one probes (Cat. No. FP1487001KT), Opal dye 690 for channel two probes (Cat. No. FP1495001KT) and Opal dye 620 for channel three probes (Cat. No. FP1497001KT). 4′,6-Diamidino-2-phenylindole (DAPI; RNAscope Multiplex Fluorescent V2 kit) was used to label nuclei, and slides were mounted with coverslips using Prolong Gold Antifade Mountant (Cat. No. P36930, Thermo Fisher Scientific). For other details of RNAscope methods, see Supplementary material.

Fluorescent images were taken using a Nikon Eclipse TE2000-S epifluorescence microscope with three channels: DAPI (nuclei), green fluorescent protein (*Gad2* channel one probe), Cyanine 5 (*Htr2c* channel two probe), TexasRed (*Slc32a1* channel three probe). Full scan images were taken at ×40 magnification with appropriate filter settings and stitched at 10% overlap, to assess brain regions for positive probe signal using ImageJ and HALO Image Analysis software (Indica Labs, Albuquerque, NM).

### Human *HTR2C* variant analysis

This study was approved by Institutional Review Boards at Baylor College of Medicine (H-32343) and Karolinska Institutet (2011/2146-31/3). Cases were enrolled in the STOP SUDEP Program at Baylor College of Medicine.^10^ We obtained a post-mortem venous blood sample or blood card from the proband and fresh venous blood samples from both parents. Genomic DNA was extracted using the Gentra Puregene Blood Kit (Qiagen) and stored at −80°C until use. DNA derived from blood cards was amplified as detailed.^39^ We performed a commercial next-generation exome sequencing on each sample (Prism Genomic Medicine, Inc., Houston, TX). The mean depth coverage was 155X, and the quality threshold was 97.6%. For the *HTR2C* gene, 100% of the coding region was covered at a minimum of 10X, *in silico* prediction of variant functional impact followed published principles^40^ built into our custom-built Mercury pipeline.^41^ Variant functional interpretation reported in ClinVar adhered to established guidelines.^42^

### Statistical methods

Statistical analyses were performed using GraphPad Prism 8 version 8.4.3 (GraphPad Software, San Diego, CA) software. Unless otherwise noted, data are presented as mean ± standard deviation, and all error bars in figures represent standard deviation. The Shapiro–Wilk normality test was performed before any parametric statistical test that assumed Gaussian distribution. If data failed the Shapiro–Wilk test, we applied the appropriate non-parametric statistical test instead. In cases of multiple comparisons, appropriate post-hoc tests were used to correct *P*-values for multiple hypothesis testing. Statistical tests and post-hoc analyses are identified when reporting *P*-values. Survival analyses, surgeries, and EEG monitoring and analyses were all performed with the experimenter blinded to genotype.

### Data availability

All supporting data in this study are available on request.

## Results

### loxTB Htr2c mutant mice show increased premature mortality

**Figure 1:**
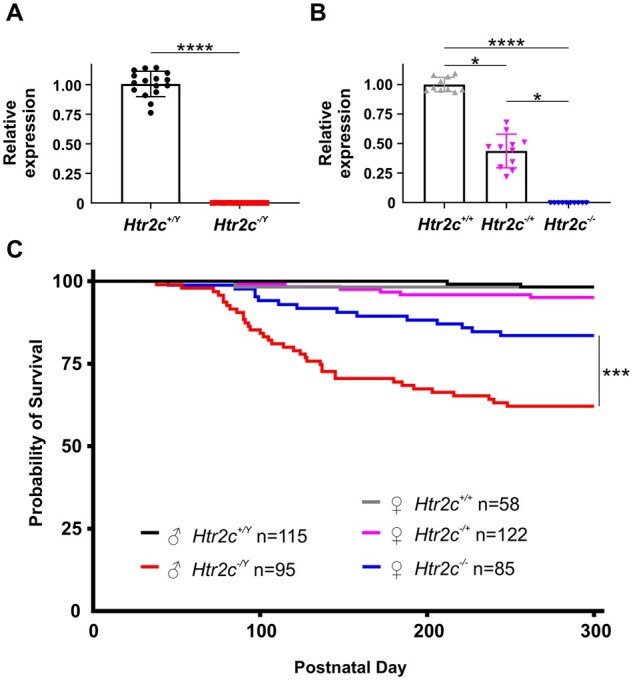
**Mice lacking 5-HT_2C_ receptors have increased premature death.** (**A**) Quantification of forebrain *Htr2c* mRNA levels in male wildtype (*n* = 16) and loxTB Htr2c mutant (*n* = 11) mice. These mutant mice have a transcriptional blocker flanked by loxP sites (loxTB) inserted between exons three and four of the X-linked *Htr2c* gene, which prevents transcription of 5-HT_2C_ receptors. These mice are functional knockouts, and we refer to male and female 5-HT_2C_-null mice as *Htr2c^−/Y^* and *Htr2c^−/−^*, respectively. Forebrain mRNA levels were normalized to the mean of wildtype expression. *Htr2c^−/Y^* mice had negligible (0.02 ± 0.04%) amplification of *Htr2c* compared to wildtype controls (*P* < 0.0001, Mann–Whitney test). (**B**) Quantification of *Htr2c* gene expression in female wildtype (*n* = 10), loxTB Htr2c *Htr2c^−/+^* (*n* = 11) and *Htr2c^−/−^* (*n* = 10) mice. A one-way ANOVA test revealed a statistical difference in *Htr2c* gene expression in female loxTB Htr2c mice (*P* < 0.0001, Kruskal–Wallis test). *Htr2c* expression in heterozygous mice was 43.68 ± 14.29% of female wildtype mice (*P* = 0.0216, Dunn’s multiple comparisons test). *Htr2c^−/−^* mice had 0% *Htr2c* expression compared to wildtype animals (*P* < 0.0001, Dunn’s multiple comparisons test). *Htr2c* expression in these mice was also significantly lower than heterozygous mice (*P* = 0.0216, Dunn’s multiple comparisons test). (**C**) Survival rates of loxTB Htr2c mutant mice are decreased compared to wildtype mice (*P* = 0.0054, Log-rank test). Male *Htr2c^−/Y^* mice (*n* = 95, red line) had increased premature mortality with a 10.00 hazard ratio when compared to *Htr2c^+/Y^* (*n* = 115, black line) mice (*P* < 0.0001, Log-rank test). Female *Htr2c^−/−^* mice (*n* = 85, blue line) had increased death with a 4.288 hazard ration compare to *Htr2c^+/+^* (*n* = 58, grey line) mice (*P* = 0.0054, Log-rank test). Male *Htr2c^−/Y^* mice had increased death compared to female *Htr2c^−/−^* mice (*P* = 0.001, Log-rank test). There was no difference in survival among *Htr2c^+/Y^*, *Htr2c^+/+^* and *Htr2c^−/+^* (*n* = 122; magenta line) mice (*P* = 0.2918, Log-rank test). * = *P* < 0.05, ** = *P* < 0.01, *** = *P* < 0.001, **** = *P* < 0.0001.

We first confirmed that loxTB Htr2c mutant mice did not express 5-HT_2C_ receptors in the brain. Currently there are no validated antibodies to determine protein expression, therefore, we utilized qPCR to assay mRNA expression. We found that male *Htr2c*^*−*^^*/Y*^ mice (*n* = 11) had undetectable (<0.02%) expression of *Htr2c* compared to wildtype (*n* = 16) littermates (*P* < 0.0001; Fig. 1A). Female *Htr2c*^*−*^^*/*^^*−*^ (*n* = 10) also were devoid of *Htr2c* expression compared to wildtype (*n* = 10) mice (*P* < 0.0001, Dunn’s multiple comparisons test; Fig. 1B). Heterozygous female mice (*Htr2c*^*−*^^*/+*^; *n* = 11) had 43.68% of *Htr2c* expression compared to female wildtype (*n* = 10) mice (*P* = 0.0216, Dunn’s multiple comparisons test; Fig. 1B). To validate the loxTB Htr2c strain as a SUDEP model, we assessed mutants for decreased survival. We found that 37.89% (36/95) of *Htr2c*^*−*^^*/Y*^ mice died prematurely (Fig. 1C, red line). This was significantly more than wildtype littermates, which had a premature mortality rate of 1.74% (2/115; *P* < 0.0001; Fig. 1C, black line) over the lifespan. The premature mortality rate was less than previously reported in male loxTB Htr2c mutant mice,^36^ but similar to that in the targeted deletion model of *Htr2c*.^35^ Since *Htr2c* is an X-linked gene and neither of these reports examined female mutant mice, we investigated the survival of female mutant loxTB Htr2c mice. We found that 16.47% (14/85; Fig. 1C, blue line) of female *Htr2c*^*−*^^*/−*^ mice died prematurely compared with only 4.92% (6/122; Fig. 1, magenta line) of *Htr2c^−^*^/+^ and 1.72% (1/58; Fig. 1C, grey line) of female wildtype mice. There was no difference in premature mortality rates of male and female wildtype or *Htr2c^−/+^* mice. Both *Htr2c^−^*^/Y^ and *Htr2c^−^*^/^^*−*^ mice had decreased survival, but *Htr2c^−/Y^* mice had a hazard ratio of 2.534, which corresponded to a mortality rate more than double that of females by PD 300 (*P* = 0.001, Log-rank test). Although male mutant mice showed substantial premature death, 62.11% of *Htr2c^−/Y^* mice (59/95) were still alive at PD 300, indicating incomplete penetrance of the SUDEP phenotype.

### loxTB Htr2c deletion causes a complex epilepsy phenotype

**Figure 2:**
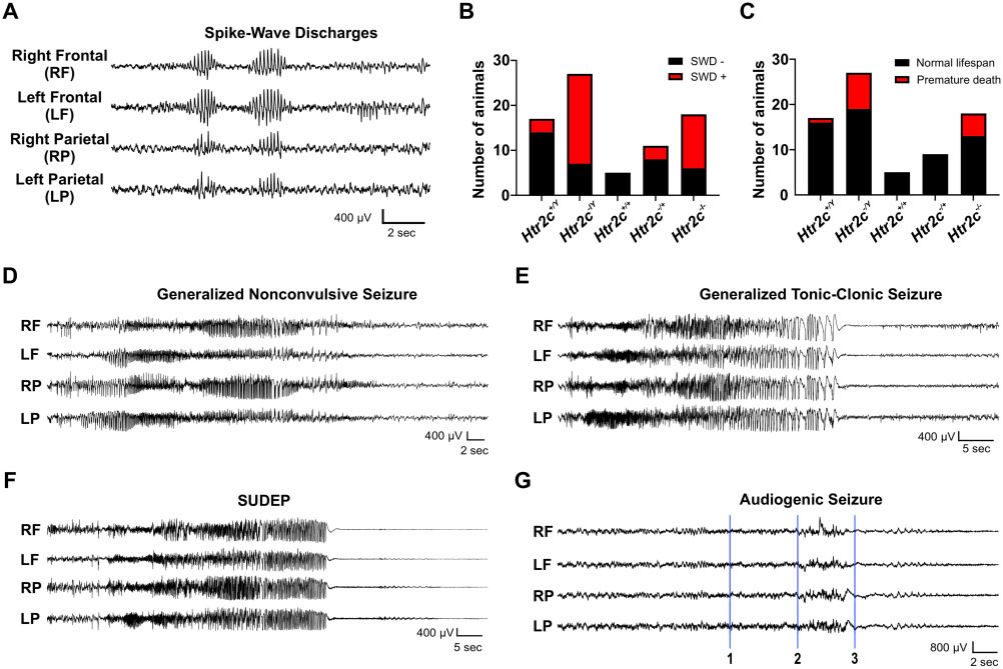
**loxTB Htr2c mutant mice have a complex epileptic phenotype with multiple seizure subtypes.** (**A**) Representative trace from a P90 male *Htr2c^−/Y^* mouse demonstrating SWDs (6–9 Hz) observed in loxTB Htr2c mice. (**B**) Histogram quantifying the presence of SWDs during video-EEG recordings in *Htr2c^+/Y^* (*n* = 17), *Htr2c^−/Y^* (*n* = 27), *Htr2c^+/+^* (*n* = 5), *Htr2c^−/+^* (*n* = 11), *Htr2c^−/−^* (*n* = 18) mice. (**C**) Histogram quantifying premature mortality in mice monitored with video-EEG. Animals that died prematurely (before PD 300) are indicated in red. These include both animals which died during video-EEG recording and those found dead in their home cage with tonic hindlimb extension. (**D**) Representative trace of a generalized nonconvulsive seizure with behavioural arrest from a P89 male *Htr2c^−/Y^* mouse. (**E**) Representative GTCS in a PD 90 male *Htr2c^−/Y^* mouse. This trace is from the same mouse in **D** approximately 24 h following the nonconvulsive seizure. (**F**) Example of a SUDEP event following a GTCS in the same PD 90 male *Htr2c^−/Y^* mouse in **D and E**. (**G**) Representative trace of an induced AGS in a PD 79 male *Htr2c^−/Y^* mouse. Alarm sound began at point 1 and mouse exhibited wild running around the recording cage at point 2. Within seconds of wild running starting, the mouse fell on its side and had tonic hindlimb extension, which resulted in death. There was no observable cortical spiking activity across any of the leads and the aberrant EEG signal during wild running can be attributed to motion artefact.

After confirming premature mortality in loxTB Htr2c mutants, we investigated whether death was due to spontaneous seizures. Previous reports indicate that 5-HT_2C_ mutants displayed spontaneous convulsive-like behaviours, but seizures were not confirmed electrographically, and their developmental onset was unspecified. Using simultaneous video-EEG recordings, we monitored brain activity in loxTB Htr2c mutants and wildtype littermates over prolonged periods from ages of PD 40–170. In young adult mutant mice (PD 40–70), the first EEG abnormality we observed in loxTB Htr2c mutants (of either sex) was frequent, bilateral spike-wave discharges (SWDs) (5–10 spikes/s, 0.5–2 s in duration, ranging from 0 to 25 per hour) similar to those seen in absence epilepsy models^43^ (Fig. 2A). These discharges appeared as early as PD 48 and were rarely seen in wildtype mice, occurring in only 17.65% (3/17; Fig. 2B) of *Htr2c^+/Y^* mice, whereas we observed SWDs in 74.07% (20/27; Fig. 2B) of *Htr2c^−/Y^* mice. Only one *Htr2c^−/Y^* died during this early stage (before PD70).

One wildtype and 13 5-HT_2C_-null mice undergoing EEG monitoring died prematurely either during a recording period or between recordings (Fig. 2C). Mutants found dead in their cage typically displayed tonic hindlimb extension similar to those incidences of SUDEP we recorded with EEG monitoring. At this age we also observed generalized non-convulsive seizures with behavioural arrest (Fig. 2D). During these electrographic seizures, mice remained immobile without any convulsive movements and rapidly regained normal behavioural at the end of the abnormal EEG discharge. Within hours to days following the onset of non-convulsive seizures, these same mice developed generalized tonic-clonic seizures (GTCS). Typically, the seizures had the same electrographic profile of the preceding non-convulsive seizures, however, they displayed both a tonic and clonic motor phase, characterized by limb clonus and followed by loss of upright posture and coordination (Fig. 2E). In some cases, convulsive seizures progressed to wild running inside the recording cage and ended suddenly with generalized EEG flattening and tonic hindlimb extension. 5-HT_2C_-null mice died within 30 s of tonic hindlimb extension (Fig. 2F). We captured such spontaneous cortical seizure/SUDEP events in one *Htr2c^−/−^* and two *Htr2c^−/Y^* mice during video-EEG recordings.

Since this postictal pattern resembled that reported following evoked AGS in adult 5-HT_2C_-null mice,^38^ we induced AGSs in three mutant mice during simultaneous EEG recording. AGS in *Htr2c^−/Y^* mice resembled those in other rodent models (wild-running followed by tonic-clonic-tonic phase, hindlimb extension and sudden death), were much shorter in duration (approximately 7–10 s) than spontaneous events, which typically lasted 20–40 s, and were uniformly immediately fatal. As in other rodent models, there was no cortical electrographic seizure activity evident during evoked AGSs (Fig. 2G), consistent with evidence that the AGS is a subcortical event triggering a brainstem mechanism of cardiorespiratory arrest.^44^

### Progressive hyperexcitability increase in adult loxTB Htr2c mutant brain

**Figure 3:**
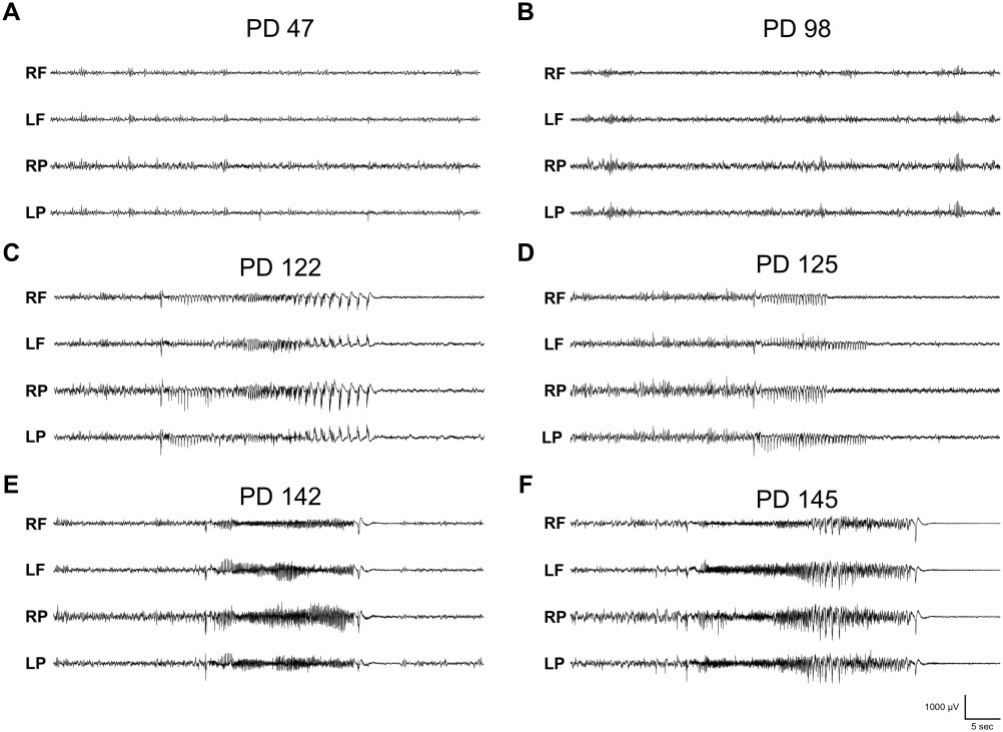
**loxTB Htr2c mutant mice show a progressive increase in hyperexcitability over their lifetime.** (**A**) Representative trace from a video-EEG recording of a PD 47 male *Htr2c^−/Y^* mouse. A few small SWDs occur periodically at this age. (**B**) Recording from the same animal at PD 98 shows more hyperactivity, including SWDs and spike discharges. (**C**) Three weeks later, at PD 122, the first monitored generalized nonconvulsive seizure appeared. (**D**) Three days later at PD 125, we observed short seizures with minor motor involvement, such as head nodding, but otherwise were nonconvulsive. (**E**) These seizures continued over the next three weeks and at PD 142 we recorded a convulsive GTCS. (**F**) Three days at PD 145, the mouse had another GTCS culminating in tonic hindlimb extension and death.

Our survival analysis indicated SUDEP first occurred after two months of age in both male and female mutants (Fig. 1), which is four to six weeks later than common monogenic developmental epileptic encephalopathy SUDEP models, such as *Scn1a, Scn8a* and *Kcna1* mutants.^45–48^ This finding prompted us to investigate whether 5-HT_2C_ mutant mice develop a slowly progressive increase in hyperexcitability and seizures during their lifespan. We recorded animals before the overt onset of convulsive seizures (<PD 75) and EEG activity was similar to wildtype littermates with only occasional interictal spikes or SWDs (Fig. 3A). However, after PD 75, we found increased abnormal hyperexcitability in 85% (23/27) of *Htr2c^−/Y^* and 78% (14/18) of *Htr2c^−/−^* mutant mice which coincided with the onset of spontaneous death in our colony (Fig. 1C). In one representative mouse (PD 98) we found SWDs with behavioural arrest, and a sharp increase in the number of interictal spikes (Fig. 3B). SWDs begins around PD 20 in most mouse models. In the same animal recorded approximately three weeks later (PD 122), we observed the first sustained generalized nonconvulsive seizure (Fig. 3C). Three days later (PD 125), we observed two short seizures that had minor motor manifestations, such as head nodding, but otherwise were nonconvulsive (Fig. 3D). Almost three weeks following this event at PD 142, additional seizures appeared that were longer in duration and progressed from nonconvulsive to GTCS (Fig. 3E). At PD 145, the mouse had a GTCS terminating in hindlimb extension and death (Fig. 3F). This representative animal exemplifies the progressive excitability increase we observed with EEG monitoring in cohorts of *Htr2c^−/Y^* mice that captured a recorded SUDEP event (*n* = 2) and those probable SUDEP cases that occurred outside of recording hours (*n* = 6). This pattern was also similar in *Htr2c^−/−^* mice undergoing EEG monitoring (*n* = 5), where we recorded one SUDEP event, and four probable SUDEP events occurred outside of recording hours.

### Restoring expression of 5-HT_2C_ receptors in Gad2-positive neurons of null mutants does not improve survival

**Figure 4:**
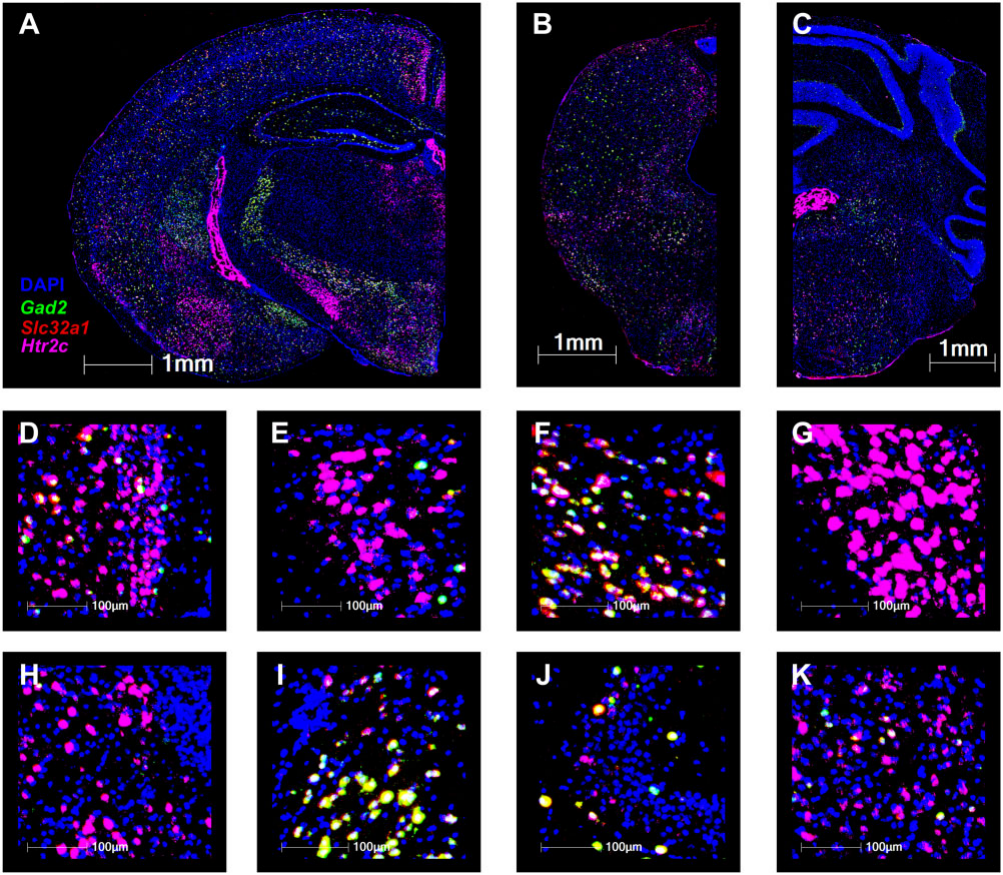
***Htr2c* mRNA is expressed throughout the forebrain and hindbrain.** (**A**) Hemi coronal forebrain section taken from a PD 84 *Htr2c^+/Y^* mouse. Tissue was stained with DAPI (blue) and processed with RNAscope FISH using probes for *Gad2* (green), *Slc32a1* (red) and *Htr2c* (magenta). (**B–C**): Hemi section from the midbrain (**B**) and medulla (**C**) of a PD 84 *Htr2c^+/Y^* mouse. Tissue was stained with DAPI (blue) and processed with RNAscope FISH using probes for *Gad2* (green), *Slc32a1* (red) and *Htr2c* (magenta). (**D–K**): ×40 insets from section shown in panel (**A**) of the following regions: retrosplenial cortex (**D**), piriform cortex (**E**), zona incerta (**F**), STN (**G**), lateral habenula (**H**), TRN (**I**), CA3a region of the hippocampus (**J**) and lateral amygdala (**K**). Separate channel images for DAPI and each of the RNAscope FISH probes are available in Supplementary Figs. 1–4.

Previous studies indicate 5-HT_2C_ receptors are expressed predominantly on GABAergic neurons.^49–51^ Moreover, when 5-HT_2C_R+ cells in the dorsal raphe nucleus are stimulated by serotonin, 5-HT_2C_R agonists, or optogenetic laser, they reduce the firing rate of principal outflow neurons, suggesting that 5-HT_2C_-expressing cells are inhibitory in this nucleus.^33^^,^^34^^,^^49^ Since available antibodies for the receptor lack specificity, we used RNAscope fluorescent *in situ* hybridization (FISH) to identify the distribution of *Htr2c* mRNA expression in mouse brain. We found that *Htr2c* showed sparse but widespread expression in both excitatory and inhibitory cells in the forebrain (Fig. 4A), midbrain (Fig. 4B) and medulla (Fig. 4C); two cortical areas showed dense expression in layers 2/3 of retrosplenial (Fig. 4D) and piriform (Fig. 4E) cortex. *Htr2c* expression was prominent in the lateral habenula (Fig. 4f) and thalamic reticular nucleus (TRN; Fig. 4G). Subthalamic structures such as zona incerta (Fig. 4H) and STN (Fig. 4I) expressed *Htr2c* mRNA at a high level. *Htr2c* signal was detected in both the CA3 region of the hippocampus (Fig. 4J) and amygdala (Fig. 4K).

Based on earlier studies, we hypothesized that a loss of 5-HT_2C_ receptors in GABAergic neurons would decrease inhibitory tone in the CNS and create a hyperexcitable state, and that allowing selective expression of 5-HT_2C_ receptors only in inhibitory neurons of a 5-HT_2C_-null mouse would restore 5-HT_2C_-mediated excitation of interneurons, thereby preserving inhibition, reducing seizures, and preventing SUDEP. We chose the glutamate decarboxylase 2 (GAD2) Cre-promoter to restore the expression of 5-HT_2C_ receptors exclusively in GABAergic neurons in the 5-HT_2C_-null mouse. GAD2 is one of two proteins utilized to synthesize GABA and is a pan-GABAergic cell marker with expression in multiple interneuron subtypes, including parvalbumin, cholecystokinin, neuropeptide Y and somatostatin.^52^

**Figure 5:**
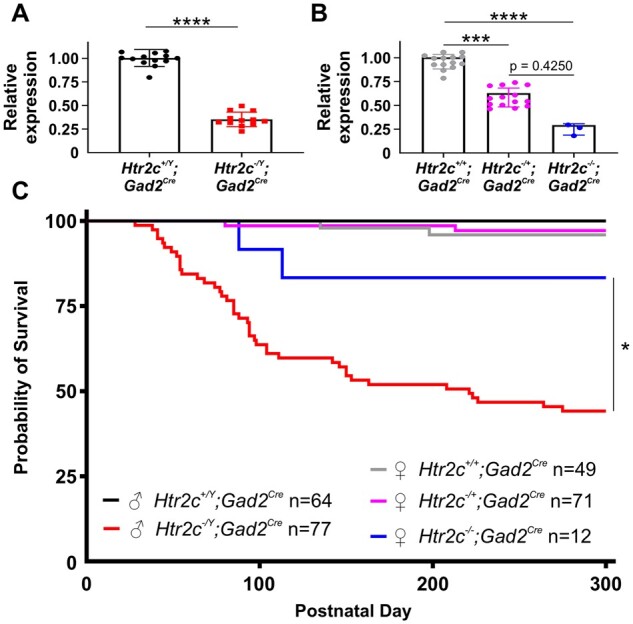
**Expression of 5-HT_2C_ receptors in Gad2-positive neurons does not prevent SUDEP.** (**A**) We bred male mice expressing Cre recombinase under the control of *Gad2* to female *Htr2c^−/+^* mice. This cross produced mice with the transcriptional blocker excised in only *Gad2*-positive neurons. Mutant mice from this cross express 5-HT_2C_ receptors in only GAD2+ neurons, and we refer to male and female mice as *Htr2c^−/Y^; Gad2^Cre^* and *Htr2c^−/−^; Gad2^Cre^*, respectively. Quantification of *Htr2c* gene expression in male Gad2^Cre^-Htr2c wildtype (*n* = 14) and mutant (*n* = 12) mice. Male *Htr2c^−/Y^; Gad2^Cre^* mice have 35.25 ± 7.71% of the *Htr2c* expression compared to wildtype littermates (*P* < 0.0001, Mann–Whitney test). (**B**) Quantification of *Htr2c* gene expression in female Gad2^Cre^-Htr2c mice. A one-way ANOVA test demonstrated there was a significant difference in gene expression among female Gad2^Cre^-Htr2c wildtype and mutant mice (*P* < 0.0001, Kruskal–Wallis test). Female *Htr2c^−/−^; Gad2^Cre^* (*n* = 3) mice have 29.30 ± 5.91% of the *Htr2c* expression of *Htr2c^+/+^; Gad2^Cre^* mice (*n* = 14; *P* = 0.0001, Dunn’s multiple comparisons test). Female *Htr2c^−/+^; Gad2^Cre^* (*n* = 14) had 62.89 ± 9.88% expression of *Htr2c* compared to female wildtype mice (*P* = 0.0003, Dunn’s multiple comparisons test). Statistical significance was not detected in expression between *Htr2c^−/+^; Gad2^Cre^* and *Htr2c^−/−^; Gad2^Cre^* (*P* = 0.4250, Dunn’s multiple comparisons test), which was most likely due to the low number of *Htr2c^−/−^; Gad2^Cre^* mice available for tissue collection. (**C**) Survival rates were different among wildtype and mutant Gad2^Cre^-Htr2c mice (*P* < 0.0001, Log-rank test). Male *Htr2c^−/Y^; Gad2^Cre^* mice (*n* = 77; red line) had increased death compared to male wildtypes (*n* = 64; black line) by P300 with a hazard ratio of 9.05 (*P* < 0.0001, Log-rank test). There was no difference in survival rates among male wildtype, female *Htr2c^−/+^; Gad2^Cre^* (*n* = 71; magenta line) and female *Htr2c^+/+^; Gad2^Cre^* (*n* = 49; grey line) mice (*P* = 0.3041, Log-rank test). We did not detect statistical differences in survival rates of female Gad2^Cre^-Htr2c mice, which was likely due to the low number of *Htr2c^−/−^; Gad2^Cre^* (*n* = 12, blue line) available to track in the colony (*P* = 0.0826, Log-rank test). * = *P* < 0.05, ** = *P* < 0.01, *** = *P* < 0.001, **** = *P* < 0.0001.

We bred female *Htr2c*^−^^/+^ mice with male mice homozygous for *Gad2^Cre^* which produced a line of mice with the loxTB element removed from the *Htr2c* gene only in *Gad2*+ cells (Gad2^Cre^-Htr2c). We verified expression of *Htr2c* by performing qPCR on forebrain tissue (Fig. 5A and B). We found that *Htr2c^−/Y^; Gad2^Cre^* mice (*n* = 12; Fig. 5A, red) express *Htr2c* mRNA at 35.25% of wildtype littermate (*Htr2c^+/Y^; Gad2^Cre^*) levels (*n* = 14; Fig. 5a, black). In female mice, *Htr2c^−/+^; Gad2^Cre^* mice (*n* = 14; Fig. 5B, magenta) and *Htr2c^−/−^; Gad2^Cre^* mice (*n* = 3; Fig. 5B, blue) expressed *Htr2c* mRNA at 62.89% and 29.30%, respectively, compared to wildtype mice (*Htr2c^+/+^; Gad2^Cre^*; *n* = 14; Fig. 5B, grey).

We then tracked the survival of mice in this line to determine if spared expression of 5-HT_2C_ receptor in *Gad2*+ neurons was sufficient to prevent SUDEP. Unexpectedly, we observed high mortality in *Htr2c^−/Y^; Gad2^Cre^* mice (55.84%; 43/77; Fig. 5C, red line) compared to wildtype littermates (0%; 0/64; *P* < 0.0001; Fig. 5C, black line). In females we recorded 16.67% mortality in *Htr2c^−/−^; Gad2^Cre^* mice (2/12; Fig. 5C, blue line) compared with only 1.49% in *Htr2c^−/+^; Gad2^Cre^* (1/67; Fig. 5C, magenta line) and 4.08% in *Htr2c^+/+^; Gad2^Cre^* (2/49; Fig. 5C, grey line) mice. These mortality rates were not lower than those observed in loxTB Htr2c male mutants (Fig. 1C). In fact, the Gad2^Cre^-Htr2c mutant mice showed death earlier than loxTB Htr2c mice. We observed only two deaths of *Htr2c^−/Y^* mice before PD 70, but in *Htr2c^−/Y^*; *Gad2^Cre^* mice we recorded 13 deaths by PD 70 (Figs. 1C and 5C). Moreover, the mortality rates of *Htr2c^−/Y^*; *Gad2^Cre^* mice (55.84%) were significantly higher than *Htr2c^−/Y^* mice (37.89%; *P* < 0.0001) (Figs. 1C and 5C). The larger gap in survival between *Htr2c^−/Y^*; *Gad2^Cre^* and *Htr2c^−/−^; Gad2^Cre^* mice could reflect a smaller number of *Htr2c^−/−^; Gad2^Cre^* mice in our colony, however, we consider it unlikely this disparity would shift survival enough to equal male *Htr2c*^*−*^^*/Y*^; *Gad2^Cre^* littermates.

### Gad2^Cre^-Htr2c mice display the same complex epileptic profile as loxTB Htr2c mice

**Figure 6:**
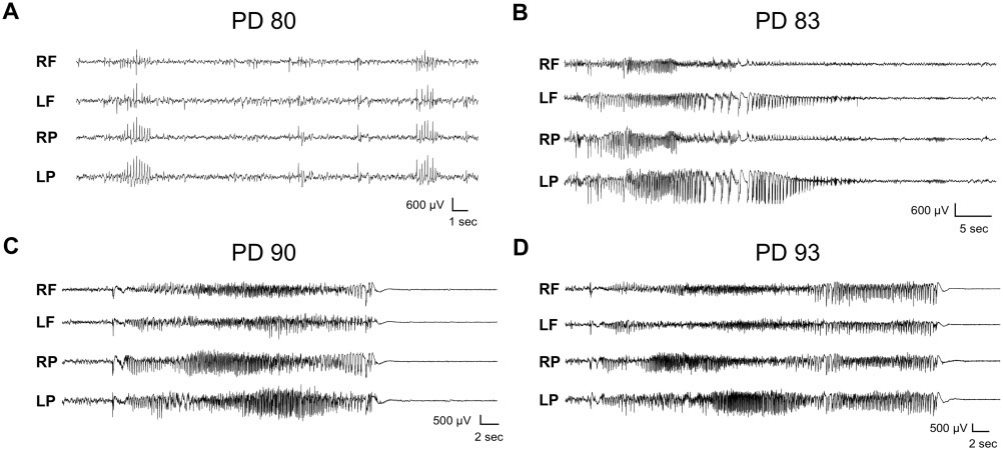
**Gad2^Cre^-Htr2c mice display the same spontaneous seizure subtypes as loxTB Htr2c mice.** (**A**) Representative trace demonstrating SWDs in a PD 80 male *Htr2c^−/Y^; Gad^Cre^* mouse. (**B**) Example of a generalized nonconvulsive seizure recorded in a PD 83 male *Htr2c^−/Y^; Gad2^Cre^* mouse. (**C**) In the same mouse as in **B**, we recorded a GTCS one week later at PD 90. (**D**) At PD 93, the same mouse died following a GTCS with tonic hindlimb extension.

Since survival did not improve, we next determined whether restoration of expression of 5-HT_2C_ receptors in GABAergic neurons had altered the epileptic phenotype of the mice. Using video-EEG recordings, we found that 75% (9/12) of *Htr2c*^*−*^*^/Y^; Gad2^Cre^* mice exhibited SWDs similar to those seen in loxTB Htr2c mice (Fig. 6A). In one representative *Htr2c*^*−*^*^/Y^; Gad2^Cre^* mouse, we recorded the same pattern of generalized nonconvulsive seizures (Fig. 6B) which then transitioned (over 7 days) into GTCS (Fig. 6C). At PD 93, the mouse had a GTCS that ended with hindlimb extension and death (Fig. 6D). Although these Gad2^Cre^-Htr2c mice spared the deletion of 5-HT_2C_ receptors in GAD2+ inhibitory neurons, the mutant mice recapitulated the same spectrum and evolution of multiple seizure types observed in the loxTB Htr2c strain. Thus, expression of 5-HT_2C_ in GAD2+ neurons was not sufficient to prevent the development of epilepsy, indicating that despite their powerful control over the epileptic phenotype, there is a minimal contribution of these receptors to 5-HT_2C_-mediated inhibitory signalling within the pathways underlying each of the various seizure subtypes.

### *HTR2C* variants are enriched in cases of human sudden unexpected death

The role of 5-HT_2C_ receptor gene variants in human premature mortality has not been explored. We reviewed our STOP SUDEP registry database of 534 patient exomes containing cases with epilepsy (*n* = 116), SUDEP (*n *= 137) and SIDS (*n* = 8). We identified 11 individuals with rare variants in *HTR2C* who died due to either definite SUDEP (*n* = 5, age range from 12 to 33 years, mean age 26.8 years), SIDS (*n* = 5, age range 2–5 months, mean age 3.5 months), or hypothermia (*n* = 1, age 34 years) (Table 1). Three of the five SUDEP cases were females (3/5; 67%), and each carried a non-synonymous (ns) single nucleotide variation in the *HTR2C* gene. In contrast, all but one of the SIDS cases were males (4/5; 80%), and carried either a ns variant (3/5), frameshift (1/5) or in-frame deletion (1/5). Review of *HTR2C* variation in gnomAD indicates that *HTR2C* is tolerant to loss of function variants (pLI = 0.04).^53^ Yet, the relative paucity of *HTR2C* variation among the 116 adult cases with presumed genetic epilepsies is intriguing and may warrant further screening in a more substantial patient sample.

**Table 1:** Summary of SUDEP and SIDS cases with *HTR2C* variation

Case	Pheno	Sex	AAD	Chr: Position	Variant†	AA change	Type	Zyg	Inh	Effect	Freq gnomAD	dbSNP	ClinVar
2273	SUDEP	M	26 Y	X: 114082728	c.512C>T	p.Ala171Val	Ns	hem	mat	likely benign	0.054% ± 0.011%	rs139581423	novel
2353	SUDEP	F	32 Y	X: 113965907	c.240G>A	p.Met80Ile	Ns	het	u	VUS	absent	novel	novel
2347	SUDEP	F	33 Y	X: 113965926	c.259G>A	p.Ala87Thr	Ns	het	u	VUS	absent	novel	novel
2354	SUDEP	F	31 Y	X: 114141802	c.1201G>A	p.Val401Ile	Ns	het	u	likely benign	absent	novel	novel
2232	SUDEP	M	12 Y	X: 114141856	c.1255A>G	p.Thr419Ala	Ns	hem	u	VUS	0.078% ± 0.013%	rs76454046	novel
2475	SIDS	M	3.5 Mo	X: 113965847	c.181_196delGTCATCATAATAATCA	p.Val61Ter	Fs	hem	u	VUS (P)	absent	novel	novel
2099	SIDS	M	3 Mo	X: 114141797	c.1196C>T	p.Pro399Leu	Ns	hem	u	VUS	absent	novel	novel
2086	SIDS	M	5 Mo	X: 114141535	c.934G>A	p.Val312Ile	ns	hem	u	VUS	absent	novel	novel
2478	SIDS	F	4 Mo	X: 114141468	c.874_876delAAG	p.Lys292del (in frame)	del	het	u	VUS	0.143% ± 0.051%	novel	novel
2474	SIDS	M	2 Mo	X: 113965886	c.220delG	p.Val74Ter	fs	hem	u	VUS (P)	absent	novel	novel
2346	EPI*	M	34 Y	X: 114141815	c.1214C>T	p.Ala405Val	ns	hem	u	VUS	absent	novel	novel

†—Reference transcript for variant calls: NM_001256760.1, GRCH38 build, AAD, age at death; Chr, chromosome; del, deletion; EPI*, patient with epilepsy that died due to hypothermia; fs, frameshift variant; het, heterozygous; Inh, inheritance; mat, maternal; Mo, months; ns, nonsynonymous; (P), possibly pathogenic; Pheno, phenotype; u, unknown inheritance; VUS, variant of uncertain clinical significance; Y, years; Zyg, zygosity.

We also reviewed the DECIPHER public copy number variation database^54^ (https://decipher.sanger.ac.uk/search?q=HTR2C#consented-patients/results, v.10.2, last accessed July 11, 2021) that houses 29 duplications and 35 deletions involving the *HTR2C* gene, ranging from 476 kb to 155.27 Mb in size, and often involving up to 60 other genes. Sex was reported in 43 cases (33 females and 10 males), and six duplications and 10 deletions were reported as either pathogenic or likely pathogenic in 12 females and four males. The most common reported phenotypes were intellectual disability, developmental delay and autism. Seizures were indicated in two females (cases 369118 and 288318), however, there were no reported cases of sudden death. The apparent absence of cases with copy number variations containing the *HTR2C* gene affected by sudden death may be due, in part, to the lack of clinical follow-up of patients referred for clinical genetic testing or due to the effect of genes co-duplicated or co-deleted within these large copy number variations.

## Discussion

Pathophysiological mechanisms of SUDEP remain unclear, and each new genetic model represents a valuable opportunity to better define the distinct predispositions that lead to sudden death and develop effective interventions. The 5-HT signalling pathway has been implicated in both human and mouse models of SUDEP,^55–57^ and continues to attract interest to clinically intervene using 5-HT releasing and reuptake inhibiting pharmacology,^58–63^ however, few monogenic models in this pathway have been systemically explored for critical cellular targets. Here, we studied the loxTB Htr2c mouse model and found that both male and female 5-HT_2C_ mutant mice display a complex epileptic phenotype with three different spontaneous seizure types and inducible AGSs. Unlike previous SUDEP mouse models, these mice experience a late-onset, progressive increase in hyperexcitability in adulthood, and a sex ratio imbalance in survival, with a mortality rate of male *Htr2c*^*−*^^*/Y*^ mice double that of female *Htr2c*^*−*^^*/*^^*−*^ mice. Although 5-HT_2C_ receptors are expressed in GABAergic interneurons where various loss-of-function mutations are thought to cause epilepsy by disinhibition, selective expression of the receptors in *Gad2*+ interneurons was not sufficient to prevent epilepsy or SUDEP.

### 5-HT_2C_ receptor knockout mice are an adult-onset SUDEP model

Two of the most studied SUDEP monogenic mouse models involve mutations of *Kcna1* and *Scn1a* genes.^45^^,^^46^^,^^48^^,^^64^^,^^65^ Each has a high seizure burden and elevated mortality in the first few weeks of life. In contrast, the EEG in 5-HT_2C_ mutant mice is unaffected before two months of age, convulsive seizures are not seen before PD 45 (Figs. 1C and 5C), and survival does not decrease until after 2 months of age. This post-pubescent onset of morbidity suggests that the contribution of 5-HT_2C_ receptors in modulating CNS excitability builds during the mouse’s lifespan. While deterioration could be contingent upon progressive neurodegeneration, no anatomical pathology in the forebrain of 5-HT_2C_ knockout mice has been reported.^66^

Another key difference between loxTB Htr2c mice and *Kcna1* and *Scn1a* SUDEP models is seizure frequency. As in humans, mouse models with high SUDEP risk typically suffer from high seizure burden. For example, *Kcna1*^*−*^^*/*^^*−*^ mice have very frequent daily seizures, and even those that survive until adulthood continue to have one to two seizures per hour throughout their life.^48^ In contrast, loxTB Htr2c mutant mice typically experience only infrequent (one to two per day) seizures. With intermittent EEG sampling, this complicated our ability to quantify absolute seizure burden. Furthermore, neither the epilepsy nor SUDEP phenotypes of loxTB Htr2c mice show full penetrance, which makes a full characterization of seizure severity and mortality risk more difficult, a feature of other SUDEP mouse models. Currently human studies have not yet determined if death is always related to the severity of the cortical seizure,^67^ or instead depends upon other intervening features, for example a lower threshold for brainstem spreading depolarization^11^ or other autonomic and cardiorespiratory factors.

### Evidence linking 5-HT signalling and SUDEP

The links between 5-HT and SUDEP have been demonstrated pharmacologically in both animal models and humans, and treatments that increase 5-HT concentration also prevent seizure-induced mortality. Fluoxetine, a 5-HT reuptake inhibitor, reduces seizure-induced respiratory arrest in both DBA/1 and DBA/2 mice without altering AGS severity.^58^ Fenfluramine, a serotonin-releasing and reuptake inhibitor, prevents respiratory arrest and death in DBA/1 mice, and decreases seizure incidence and severity as well.^58^ Fenfluramine and fluoxetine reduced seizure frequency in patients with Dravet syndrome.^60–63^ Although fenfluramine is a weak 5-HT_2C_ agonist, its metabolite, norfenfluramine, has high affinity for 5-HT_2C_ receptors.^68^ Other 5-HT_2C_ agonists, including lorcaserin and trazodone, have also shown effectiveness in reducing seizures in a Zebrafish model of Dravet syndrome.^69^ Interestingly, lorcaserin also suppresses SWDs in a rat genetic absence epilepsy model.^70^ Post-ictal generalized EEG suppression (PGES) is a possible biomarker for SUDEP risk in human patients.^71^ Although the mechanisms that underlie PGES remain unclear, recent evidence has demonstrated that increased 5-HT or 5-HT neuron activity reduces PGES in experimental models.^55^^,^^72^ Although our study was not designed to fully characterize PGES in 5-HT_2C_-null mice, we noted reduced EEG signal amplitude following non-fatal GTCS events (Figs. 2E and 4C). Future studies will investigate this potential biomarker for SUDEP risk in loxTB Htr2c mutant mice. Finally, in a post-mortem study of brainstem tissue, patients that died of SUDEP had decreased expression of tryptophan hydroxylase, the rate-limiting step in 5-HT synthesis, and the 5-HT transporter compared to epilepsy controls.^73^ Our findings, when paired with these supporting data, highlight 5-HT_2C_ receptors as an important molecular target within the 5-HT signalling pathway capable of modulating seizures and premature death.

### Sex ratio differences in SUDEP mortality

In human SUDEP, male sex confers a higher risk for mortality,^6^^,^^74^ but few SUDEP mouse models to date show a sexual dimorphism for survival. One recent study reported a sex difference in mortality of *Scn1a^+/^*^*−*^ mice, however, in this study, female mutants had increased mortality.^75^ Our data indicate that mortality in male *Htr2c*^*−*^^*/Y*^ mice is elevated compared to female *Htr2c*^*−*^^*/*^^*−*^ mice (Fig. 1). The mechanisms contributing to the sex difference are obscure but could be hormonal. Ovarian sex hormones, including oestrogens and progesterones, play an important role in modulating CNS excitability.^76^ Interestingly, female rodents have increased concentrations of 5-HT and 5-hydroxyindolacetic acid, the primary metabolite of 5-HT.^77–80^ Ovarian hormones upregulate 5-HT synthesis and turnover, which are markers of 5-HT activity.^77^^,^^78^^,^^81^^,^^82^ In addition, hormone replacement therapy in ovariectomized rodents activates the 5-HT system.^83–87^ A Swedish population-based cohort study reported a higher SUDEP incidence in males, and the sex difference was most prominent in the youngest cohort,^74^ while the overwhelming majority of SUDEP cases in women occurred after the age of 50.^74^ These data suggest that SUDEP risk for women increases with age, which may be related to post-menopausal hormonal changes. Future studies are needed to explore mechanisms underlying SUDEP sex differences in 5-HT_2C_ mutants and other SUDEP models.

### Epilepsy in 5-HT_2C_ mutant mice is not solely due to disinhibition mediated by GAD2+ interneurons

5-HT_2C_ receptors are widely, but distinctly, distributed in the CNS.^29–31^ Liu et al.^88^ reported that 5-HT_2C_ receptors were localized to GABAergic neurons in layer IV of the prelimbic prefrontal cortex in rat brain and are preferentially expressed on parvalbumin-positive cells compared to those expressing calbindin or calretinin. A study of 5-HT_2C_ receptors in the anterior raphe nuclei showed that these receptors are located on GAD1-positive GABAergic interneurons.^89^ Our RNAscope data confirm overlapping interneuron co-expression in many of these areas, since an overwhelming number of cells co-expressed *Htr2c* and the GABAergic marker *Gad2* and/or *Slc32a1* (Fig. 4). These data suggest that 5-HT_2C_ receptors play a role in a negative feedback loop by stimulating local GABAergic interneurons. Boothman et al.^33^ demonstrated that application of a 5-HT_2C_ agonist, WAY 161503, decreased 5-HT neuron firing, and this effect could be blocked with 5-HT_2C_ antagonists. This study also reported increased Fos expression in GABAergic neurons after WAY 161503 exposure suggesting 5-HT_2C_ receptors activated these neurons.^33^ Another group used an optogenetic probe localized to 5-HT_2C_-expressing GAD2+ neurons to test the effect of 5-HT_2C_ receptors on dorsal raphe activity and found that light stimulation activated these GABAergic neurons and inhibited 5-HT neurons.^34^ These data confirm a role for 5-HT_2C_ receptors in inhibitory interneuron signalling.

We therefore hypothesized that loss of 5-HT_2C_ receptors that activate GABAergic neurons provides a parsimonious explanation for epilepsy and SUDEP in loxTB Htr2c mice, however, in the Gad2^Cre^-Htr2c model with selective expression only in *Gad2*+ interneurons, we observed identical EEG abnormalities and spontaneous seizures, and premature mortality was increased rather than blunted (Fig. 5C). These data argue against a major role of 5-HT_2C_-linked disinhibition underlying the epilepsy/SUDEP phenotype and suggest that 5-HT_2C_ receptors expressed in non-GABAergic neurons must play a more complex role in controlling CNS network excitability. Although most cells expressing 5-HT_2C_ receptors are GABAergic, one study reported that 27% of 5-HT_2C_-expressing cells are non-GABAergic^90^ which may explain the complex seizure phenotype and sudden death in the Gad2^Cre^-Htr2c model. Future experiments that identify and selectively express 5-HT_2C_ receptors in them may provide insight into critical pathways for each of these phenotypic elements.

### *HTR2C* variants in SUDEP and SIDS populations

The sex-linked premature mortality in our model prompted us to perform the first variant analysis of *HTR2C* in a clinical exome sequencing cohort and in publicly available human SUDEP and SIDS databases. Although SIDS is a diagnosis of exclusion and by definition does not include cases with known epilepsy, there is neuropathological evidence of hippocampal malformations in many of these cases, suggesting increased risk of unrecognized seizure activity.^91–93^ A recent report indicates that up to 35% of SIDS cases may be due to genetic diseases including 9% of cases with likely pathological variants in ion channelopathy-associated genes.^94^ In addition, Brownstein et al.^95^ found likely pathogenic *SCN1A* variants in SIDS cases. Determining if there are *HTR2C* variants in these databases is important since disrupted serotonergic signalling is implicated in both SUDEP and SIDS.^57^^,^^96^^,^^97^ We found a sufficient enrichment of non-synonymous variants to warrant a larger scale human association study in the future. In our small cohort, non-synonymous variants dominate in male SIDS cases (4/5, 80%) whereas 67% (4/6) of SUDEP cases were older females. It is tempting to speculate that variants in SIDS were more detrimental and that early mortality precluded the appearance of seizures. Sex differences may also arise by imprinting of the variant allele. However, given the small sample size, and unclear functional effects of the variants, it is premature to draw human parallels, and collaborative large-scale studies of 5-HT receptor variation in human SUDEP and SIDS cohorts are needed.

In summary, our studies of loxTB Htr2c mice clearly reveal a SUDEP model with increased mortality beginning in adulthood and, to our knowledge, the first evidence for a gene-linked male sex predominance in SUDEP incidence in a monogenic animal model. We also demonstrate that whereas absence of this receptor causes epilepsy, selectively restoring 5-HT_2C_ receptors only in interneurons is insufficient to prevent seizures and premature death in mice. This finding, consistent with the generally held view of serotonin as a central neuromodulator, implies a more complex network imbalance where dysmodulation of excitatory cells may play a more prominent role in epileptogenesis despite a relatively intact expression of 5-HT_2C_ receptors on GABAergic inhibitory cells. Future studies using selective expression in excitatory neurons may confirm this mechanism, or possibly suggest that 5-HT_2C_ receptors require precise rebalancing among different interneuron subtypes.

## Supplementary material

Supplementary material is available at *Brain Communications* online.

## Supplementary Material

fcab149_Supplementary_Data

## Abbreviations

AGS =: audiogenic seizure
DAPI =: 4′,6-diamidino-2-phenylindole
FISH =: fluorescent *in situ* hybridization
5-HT =: serotonin
GAD2 =: glutamate decarboxylase 2
GTCS =: generalized tonic clonic seizure
s.c. =: subcutaneous
PD =: postnatal day
PGES =: post-ictal generalized EEG suppression
qPCR =: quantitative PCR
SIDS =: sudden infant death syndrome
SUDEP =: sudden unexpected death in epilepsy
SWD =: spike-wave discharge
