# Supplementary material for: X-linked serotonin 2C receptor is associated with a non-canonical pathway for sudden unexpected death in epilepsy
Source: Brain Commun. 2021 Jul 9;3(3):fcab149. doi: 10.1093/braincomms/fcab149 (PMC8361391; doi:10.1093/braincomms/fcab149)
Supplement: fcab149_Supplementary_Data [file fcab149_supplementary_data.pdf]

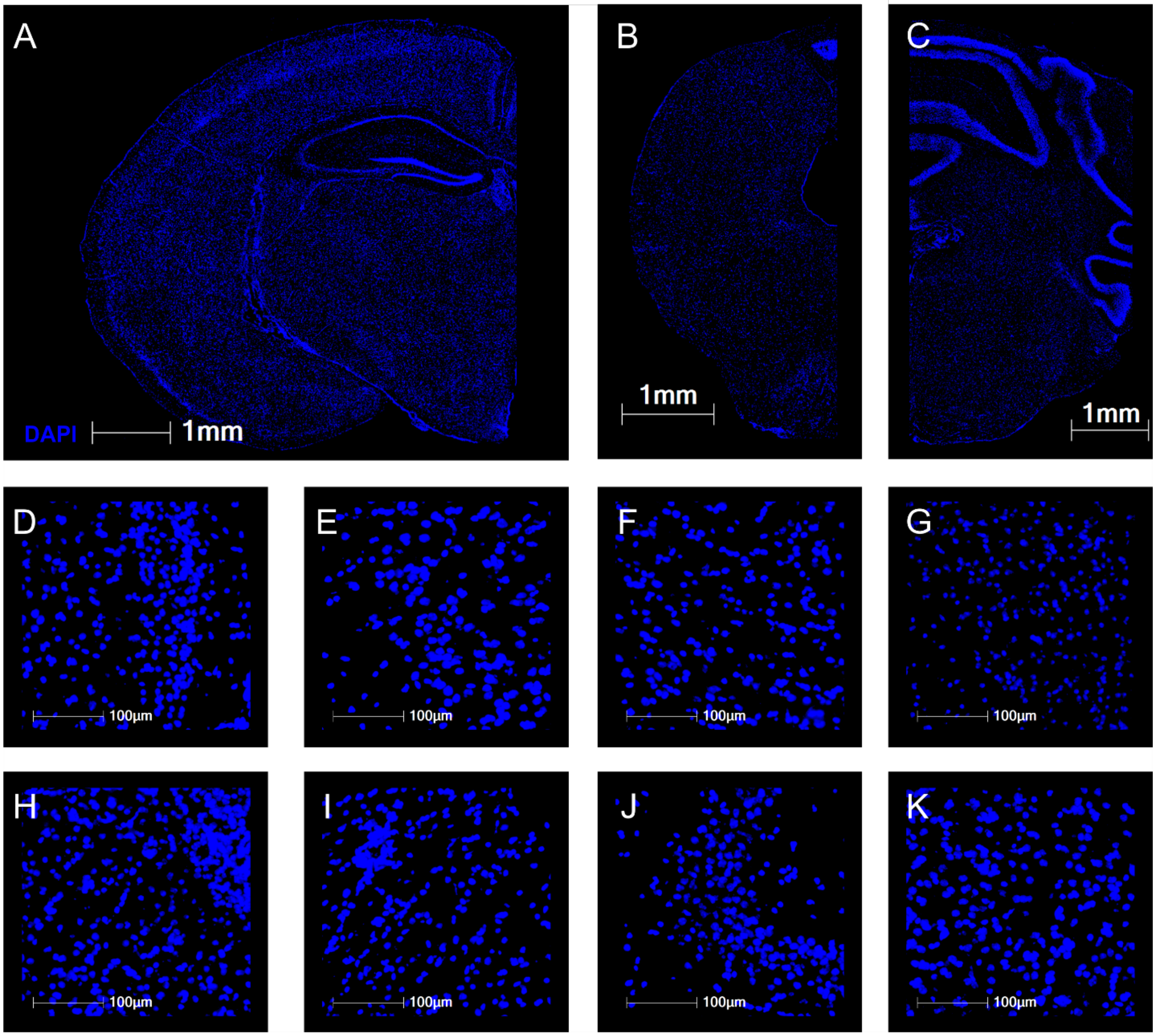

Supplementary Figure 1. Single DAPI channel images of tissue processed for RNAscope. a) Hemi coronal forebrain section taken from a PD 84 *Htr2c*$^{+/Y}$ mouse. Tissue was stained with DAPI (blue). **b-c):** Hemi section from the midbrain (**b**) and medulla (**c**) of a PD 84 *Htr2c*$^{+/Y}$ mouse. Tissue was stained with DAPI (blue). **d-k):** 40X insets from section shown in panel (a) of the following regions: retrosplenial cortex (**d**), piriform cortex (**e**), zona incerta (**f**), STN (**g**), lateral habenula (**h**), TRN (**i**), CA3a region of the hippocampus (**j**), and lateral amygdala (**k**).

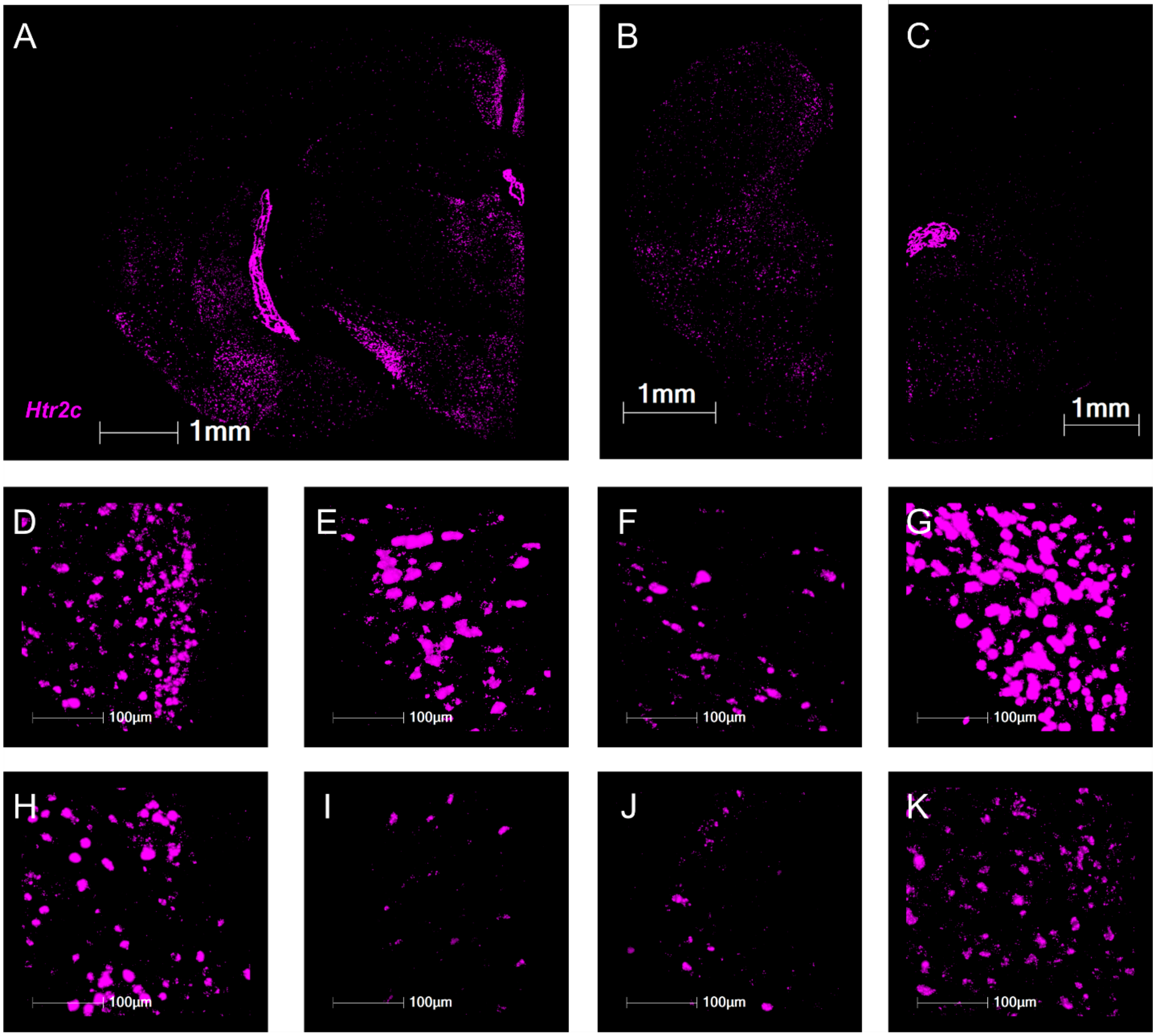

Supplementary Figure 2. Single CY5 channel images of tissue processed with *Htr2c* RNAscope probes. a) Hemi coronal forebrain section taken from a PD 84 $Htr2c^{+/Y}$ mouse. Tissue was processed with RNAscope probes for *Htr2c* (magenta). **b-c):** Hemi section from the midbrain (**b**) and medulla (**c**) of a PD 84 $Htr2c^{+/Y}$ mouse. Tissue was processed with RNAscope FISH using probes for *Htr2c* (magenta) **d-k):** 40X insets from section shown in panel (a) of the following regions: retrosplenial cortex (**d**), piriform cortex (**e**), zona incerta (**f**), STN (**g**), lateral habenula (**h**), TRN (**i**), CA3a region of the hippocampus (**j**), and lateral amygdala (**k**).

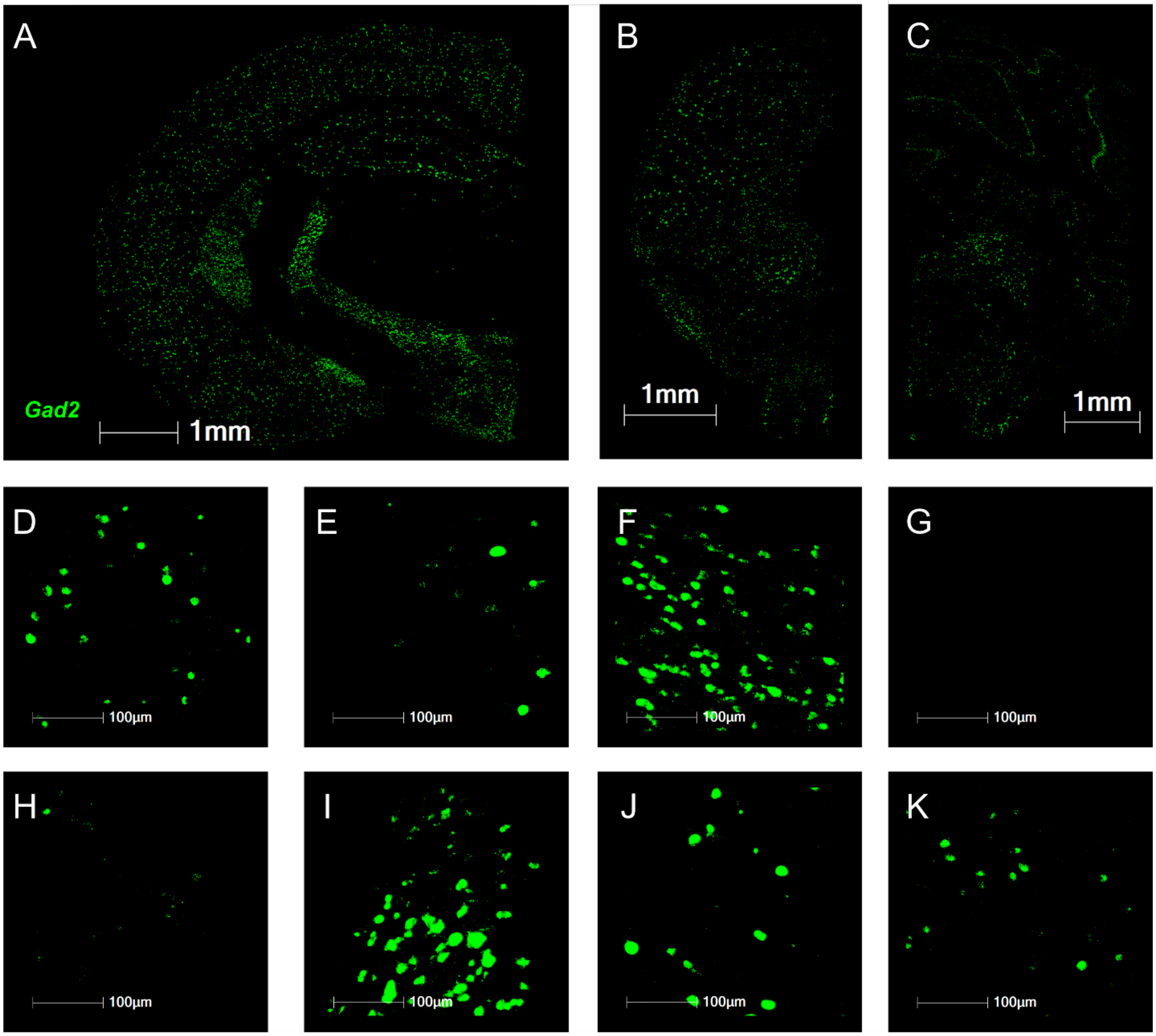

Supplementary Figure 3. Single GFP channel images of tissue processed with *Gad2* RNAscope probes. a) Hemi coronal forebrain section taken from a PD 84 *Htr2c*$^{+/Y}$ mouse. Tissue was processed with RNAscope probes for *Gad2* (green). **b-c):** Hemi section from the midbrain (**b**) and medulla (**c**) of a PD 84 *Htr2c*$^{+/Y}$ mouse. Tissue was processed with RNAscope FISH using probes for *Gad2* (green). **d-k):** 40X insets from section shown in panel (a) of the following regions: retrosplenial cortex (**d**), piriform cortex (**e**), zona incerta (**f**), STN (**g**), lateral habenula (**h**), TRN (**i**), CA3a region of the hippocampus (**j**), and lateral amygdala (**k**).

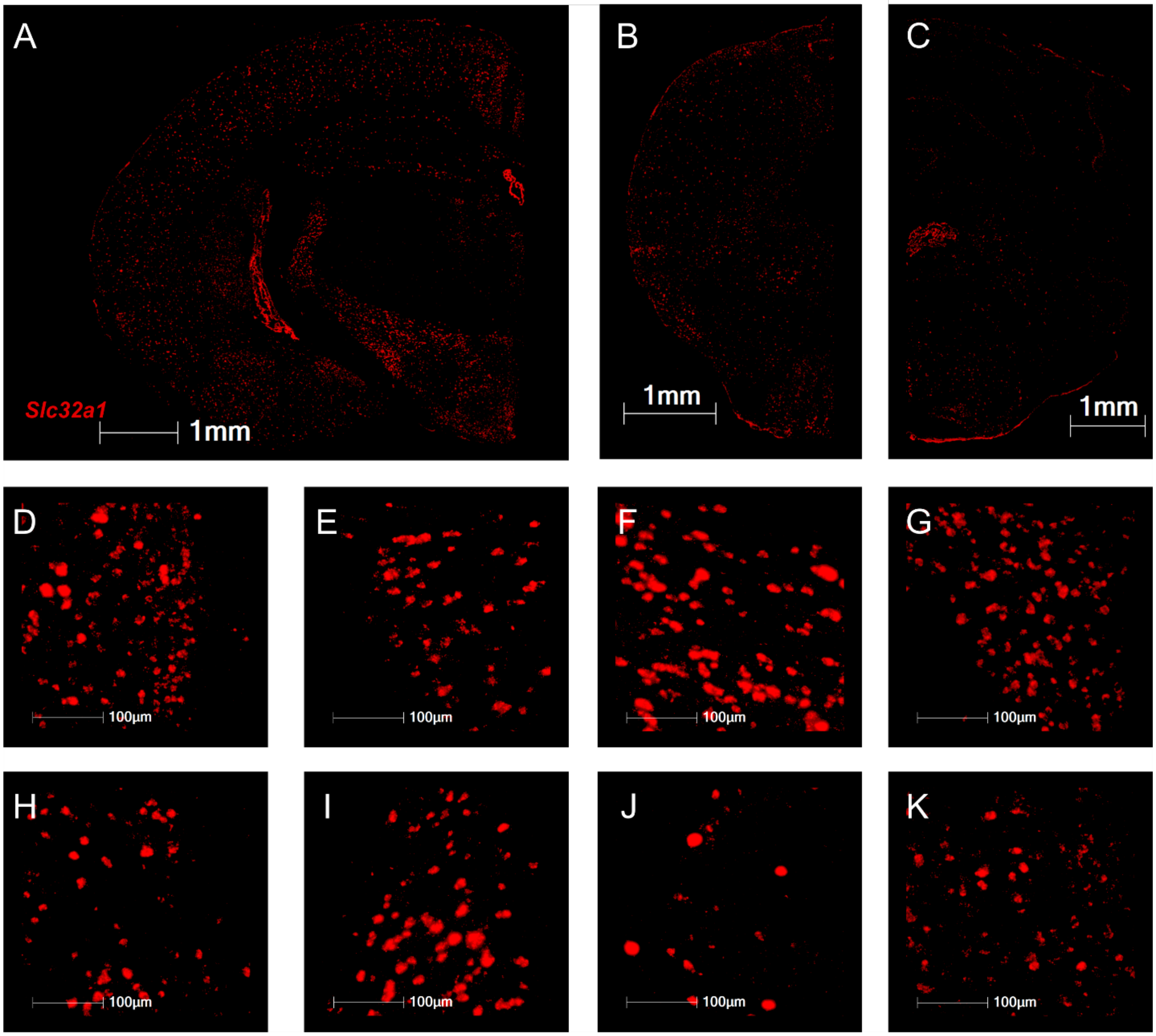

Supplementary Figure 4. Single TexasRed channel images of tissue processed with *Slc32a1* RNAscope probes. a) Hemi coronal forebrain section taken from a PD 84 *Htr2c*$^{+/Y}$ mouse. Tissue was processed with RNAscope probes for *Slc32a1* (red). **b-c):** Hemi section from the midbrain (**b**) and medulla (**c**) of a PD 84 *Htr2c*$^{+/Y}$ mouse. Tissue was processed with RNAscope FISH using probes for *Slc32a1* (red). **d-k):** 40X insets from section shown in panel (a) of the following regions: retrosplenial cortex (**d**), piriform cortex (**e**), zona incerta (**f**), STN (**g**), lateral habenula (**h**), TRN (**i**), CA3a region of the hippocampus (**j**), and lateral amygdala (**k**).

Supplementary material

Additional Video-EEG recording criteria

Mice were recorded with simultaneous video for 24 hours a day over 7-10 day periods. Afterward mice were returned to their home cage for 2-7 days before another set of recordings were performed. If we observed seizures, we extended the monitoring for a period of up to 21 days in compliance with institutional guidelines. If EEG signals became unstable due to implant damage or loss, video-EEG monitoring was discontinued. Although there was an increased mortality rate in mutant loxTB Htr2c mice, the seizure events were rarer than other epileptic strains. Due to these factors, we increased the numbers of mutant mice being monitored with EEG recordings. Since the gene is X-linked, there were no wildtype littermates for female 5-HT_{2C}-null mice. We also observed very little premature death in the female wildtype animals and therefore decided to record EEG from more *Htr2c*$^{+/-}$ and *Htr2c*$^{-/-}$ mice.

Seizure type criteria

Seizures were assessed using standard semiological criteria correlating electrographic patterns with episodic motor behaviors such as tonic clonic movements during generalized convulsions, behavioral arrest during spike-wave discharges, and wild running during audiogenic seizures. All seizures were visually inspected and manually annotated.

qPCR analysis

Threshold cycle (C_t) values were measured for *Htr2c* and *Gapdh* for each sample and replicate in the same well. The difference between these two values (ΔC_t) for each animal was calculated and averaged between replicates. ΔC_t values for each animal were normalized to the mean ΔC_t of wildtype mice of the same sex ($\Delta\Delta C_t$), which was then converted to relative quantification (RQ)

values with the formula: RQ = $2(-\Delta\Delta C_t)$. Data was analyzed with QuantStudio Design and Analysis software (Applied Biosystems).

RNAscope tissue processing

After removing tissue from -80 °C, slides were covered with room temperature 4% paraformaldehyde fixative for 1 hour, dehydrated in ethanol, and baked at 60 °C for 1 hour. Sections were pretreated with hydrogen peroxide to quench endogenous peroxidase activity and protease IV to allow probes to access RNA targets. Channel two and three probes were diluted into channel one probes at a 1:1:50 ratio then placed on tissue sections for probe hybridization in a humidified incubator at 40 °C for 2 hours. Then slides were placed into 5X sodium saline citrate overnight. The following day probes were amplified, conjugated to a channel-specific Horseradish peroxidase (HRP), and labeled with Akoya labeled using Akoya Biosciences (Marlborough, MA) Opal fluorescent dyes at a 1:1500 dilution. After fluorescent dye was applied to each channel, an HRP blocker was applied to quench any residual HRP reaction. Once the slides were mounted with coverslips, they were placed in a dark container to dry overnight. The following morning slides were transferred to a slide box and stored at 4 °C for at least 48 hours before imaging on a microscope.
